# Dynamics of water-mediated interaction effects on the stability and transmission of Omicron

**DOI:** 10.1038/s41598-023-48186-2

**Published:** 2023-11-28

**Authors:** Naila Zaman, Nousheen Parvaiz, Fouzia Gul, Rimsha Yousaf, Kainat Gul, Syed Sikander Azam

**Affiliations:** Computational Biology Lab, National Center for Bioinformatics (NCB), Quaid-i-Azam University, Islamabad, 45320 Pakistan

**Keywords:** Computational biology and bioinformatics, Drug discovery

## Abstract

SARS-Cov-2 Omicron variant and its highly transmissible sublineages amidst news of emerging hybrid variants strengthen the evidence of its ability to rapidly spread and evolve giving rise to unprecedented future waves. Owing to the presence of isolated RBD, monomeric and trimeric Cryo-EM structures of spike protein in complex with ACE2 receptor, comparative analysis of Alpha, Beta, Gamma, Delta, and Omicron assist in a rational assessment of their probability to evolve as new or hybrid variants in future. This study proposes the role of hydration forces in mediating Omicron function and dynamics based on a stronger interplay between protein and solvent with each Covid wave. Mutations of multiple hydrophobic residues into hydrophilic residues underwent concerted interactions with water leading to variations in charge distribution in Delta and Omicron during molecular dynamics simulations. Moreover, comparative analysis of interacting moieties characterized a large number of mutations lying at RBD into constrained, homologous and low-affinity groups referred to as mutational drivers inferring that the probability of future mutations relies on their function. Furthermore, the computational findings reveal a significant difference in angular distances among variants of concern due 3 amino acid insertion (EPE) in Omicron variant that not only facilitates tight domain organization but also seems requisite for characterization of mutational processes. The outcome of this work signifies the possible relation between hydration forces, their impact on conformation and binding affinities, and viral fitness that will significantly aid in understanding dynamics of drug targets for Covid-19 countermeasures. The emerging scenario is that hydration forces and hydrophobic interactions are crucial variables to probe in mutational analysis to explore conformational landscape of macromolecules and reveal the molecular origins of protein behaviors.

## Introduction

Viruses constantly adapt and evolve through mutations giving rise to new variants some of which are resistant to vaccines and have high transmissibility rate^1,2^. SARS-CoV-2, the virus responsible for Covid19 pandemic has also formed genetic lineages over time^3^. These lineages vary in their characteristics affecting the spread and severity of infection caused by the virus and possible effective treatment options that can be availed^4,5^. Currently, all the active lineages of SARS-CoV-2 are classified as part of Omicron variant which is threatening to worsen the transmissibility rate, diagnosis, re-infection, and performance of vaccines due to multiple mutations^6,7^. The world is acknowledging Omicron as far more transmissible and agile in evading immune defense systems than previous mutated variants, especially the Delta variant^8–11^. Variants that emerged in the following years of pandemic and are categorized as variants of concern (VoCs) by WHO (https://www.who.int/activities/tracking-SARS-CoV-2-variants) are Alpha (B.1.1.7 lineage), Beta (B.1.351), Gamma (P.1), Delta (B.1.617.2), and now Omicron (BA.1, BA.1.1 and BA.2).

Delta variant has predominated in global epidemiology of SARS-CoV-2 prevailing in 99.8% of analyzed sequences^12^ uploaded to the GISAID database (https://www.gisaid.org/hcov19-variants/). Delta variant acquired 10 mutations in spike protein whereas Omicron has exhibited 32 mutations including 15 mutations in receptor binding domain (RBD), 3 deletions, and one insertion^7,13–15^. Different studies have reported the effect of mutations on binding between the ACE2 receptor and spike protein leading to disparity in the rate of transmission and virulence over the period of time^16–18^.To study the effect of individual mutations on binding affinity of ACE2, Starr et al*.* conducted a systematic experimental study, which categorized 9 mutations (S371L, S373P, S375F, K417N, G446S, E484A, G496S, Q498R, Y505H) as decreasing and other 6 mutations (G339D, N440K, S477N, T478K, Q493K, N501Y) involved in increasing deleterious effects of the variant due to enhanced binding affinity^19^. Moreover, the higher binding affinity of recently reported mutations D614G have been linked with an increased rate of transmission and viral load in Covid19 patients^20^ whereas deleted residues in Delta variant 157–158 were connected with antibody escape^21^. Furthermore, commonly found mutations among other variants particularly Omicron that exhibited significant structural changes in combination and are particularly linked with cell tropism affecting receptor recognition patterns include N679K, N501Y, and P681H^22,23^. Additional mutations not only appeared at RBD that are directly involved in cell entry of SARS-CoV-2 resulting in enhanced attachment to its receptor but also played a critical role in priming of the protein^24–26^. These two major events; prefusion to the postfusion stage of the viral life cycle were drastically effected by subsequent mutations in sub-domains of spike protein as mentioned in multiple studies^24,27,28^.Existing vaccines have shown reduced efficacy against Omicron variant due to its immune evasion strategy. In a study conducted by Pulliam et al*.* it was established that Omicron variant is associated with increase in reinfection risk coefficient and decrease in primary infection risk coefficient^29^.

Therefore, to unveil the molecular basis for a higher number of mutations in each wave, this study compares the dynamic differences between SARS-CoV-2 variants of concern and wild-type with specific interest on the Omicron variant by employing MD simulations. These comparisons were carried out on spike protein of all VoCs as a trimer, a monomer, and the receptor binding domain (RBD) alone. The findings highlight the significant role of hydration forces in providing stability with particular emphasis on mutations-induced intra/intermolecular changes in solution and molecular determinants responsible for relative binding affinities. Moreover, the 3 amino acid insertion (EPE) in Omicron BA.1^15,30,31^ is proposed to hold a precarious position in forecasting the impact of bulk mutations on conformational dynamics. Complete coherence of structural mutations and outcomes of MD simulations further strengthens the possible relation between mutations and viral fitness that will significantly aid in understanding the molecular basis of additional mutations in new variants. Furthermore, the research data also tends to examine the role of hydration forces in guiding the structure, stability, function, and dynamics of VOCs, a subject that yet remains unexplored in present studies.

## Materials and methods

### Predicting the impact of multiple point mutations

DynaMut, a comprehensive suite for protein motion and flexibility analysis^32^ was used to explore molecular consequences of mutations in different VoCs. The tool implements Normal Mode Analysis (NMA) using ENCoM and Bio3D approaches to analyze protein motions. It also considers the vibrational entropy changes that influence protein dynamics and stability. For protein dynamics analysis, the structure of wild-type (WT) spike protein was given as input. The ‘Mutation list’ option was then used to upload the list of mutations for each VoCs for batch processing. The server then uses the consensus prediction method, which includes performance metrics, and machine-learning algorithms namely; Random forest and step-wise reedy approach to explore the impact of protein mutations on interactions with other residues^33^.

### Structure modeling of mutants

SARS-CoV-2 ACE2 complex (PDB ID: 7df4), was selected as a suitable template for structure modeling of WT. Three-Dimensional (3D) structure of WT and all variants of Covid19 were generated using SWISS-MODEL (https://swissmodel.expasy.org/). SWISS-MODEL is an automated server that predicts the 3D structure of proteins using homology modeling techniques^34,35^. Mutated amino acid sequences for all variants were entered and the template was selected for each variant respectively. PDB ID: 7a94 showed the highest homology to all input sequences and was selected as a template to generate 3D structures of Alpha (B.1.1.7), Beta (B.1.351), Gamma (P.1), Delta (B.1.617.2), and Omicron (B.1.1.529).

Alpha variant has 3 deletions (H69, V70, and Y144) and 7 single mutations namely (N501Y, A570D, D614G, P681H, T7161, S982A, and D1118H)^36,37^. Whereas, the Beta variant is associated with several mutations in spike, including 3 deletions (L241, L242, and A243) and 8 single mutations (L18F, D80A, D215G, K417N, E484K, N501Y, D614G, and A701V)^38–40^. Deletion at H69 and V70 positions is not present in Beta variant. However, in the Gamma variant, several single mutations (L18F, P26S, T20N, D138Y, R190S, K417T, E484K, N501Y, D614G, H655Y, and T1027I) are observed but no deletions have been reported^37,41,42^. Furthermore, 6 spike mutations in Delta variant are observed at positions (T19R, L452R, T478K, D614G, P681R, and D950N). In addition to these, 2 deletions (E156 and F157) are also associated with Delta variant^39,42^. Titers against the Delta variant were reported to be around twofold lower in comparison to Alpha and WT^43–45^. Lastly, the Omicron variant that is the highly mutated Covid19 variant^7,15^ includes 6 deletions (H69, V70, Y144, G142, V143, and N211), 29 single point mutations i-e: A67V, T95I, Y145D, K417N, S477N, T478K, E484A, N501Y, D614G, H655Y, P681H, G339D, S371L, S373P, S375F, N440K, G446S, Q493R, G496S, Q498R, Y505H, T547K, N679K, N764K, D796Y, N856K, Q954H, N969K, and L981F and one insertion at position 214EPE^9,14^. Mutations in Omicron are not evenly distributed such as there are 15 mutations that occurred at the RBD and 10 mutations occurred at the Receptor Binding Motif (RBM), which interact directly with the ACE2 receptor^46,47^. Omicron spike is 3 amino acids less in comparison to the WT^15,48,49^. Mutations in Alpha, Beta, Gamma, and Omicron are listed in Table 1 and divided into 2 sections: (1) refers to the shared mutations among all VoCs and (2) refers to the novel mutations observed in particular variants.

**Table 1:** Mutations in Alpha, Beta, Gamma, Delta, and Omicron.

Alpha, V1 (B.1.1.7)	Beta, V2 (B.1.351)	Gamma, V3 (P.1)	Delta (B.1.617.2)	Omicron (B.1.1.529)
Shared mutations				
S:H69-	S:L18F	S:L18F	S:L452R	S:A67V
S:V70-	S:L241-	S:P26S	S:T478K	S:H69-
S:Y144-	S:L242-	S:K417T	S:D614G	S:V70-
S:N501Y	S:A243-	S:E484K	S:P681R	S:T95I
S:D614G	S:K417N	S:N501Y	S:D950N	S:Y144-
S:P681H	S:E484K	S:D614G		S:Y145D
S:D1118H	S:N501Y	S:H655Y		S:K417N
	S:D614G	S:T1027I		S:S477N
	S:A701V			S:T478K
				S:E484A
				S:N501Y
				S:D614G
				S:H655Y
				S:P681H
Other mutations				
S:A570D	S:D80A	S:T20N	S:T19R	S:G142-
S:T7161	S:D215G	S:D138Y	S:E156-	S:V143-
S:S982A		S:R190S	S:F157-	S:N211-
				S:G339D
				S:S371L
				S:S373P
				S:S375F
				S:N440K
				S:G446S
				S:Q493R
				S:G496S
				S:Q498R
				S:Y505H
				S:T547K
				S:N679K
				S:N764K
				S:D796Y
				S:N856K
				S:Q954H
				S:N969K
				S:L981F

### Molecular dynamics simulations

MD simulations that play a significant role in investigating the dynamic behavior and conformational space of proteins, were carried out to compare the dynamic differences between SARS-CoV-2 VoCs and wild-type. These comparisons were drawn through 2800 ns MD simulations in total on spike protein as a trimer, a single monomer and the receptor binding domain (RBD) alone. The system of the complexes was prepared using the antechamber program of AMBER (Assisted model building with energy refinement) to generate the topology and parameter files^50,51^. The topology of these systems was generated using the ff14SB force field^52^ whereas the system was solvated by placing complexes in a cubic box of 15 Å TIP3P water box^53,54^ with 18.774 Å × 18.774 Å × 18.774 Å solvent unit box dimensions. Neutralization of all the system was achieved by addition of Na + ions to it. For RBD and monomer omicron variants 42 Na + ions were added while for trimers 20 Na + ions were added. All systems then underwent the steps of minimization followed by heating and equilibration. Minimization was carried out for 1000 cycles with constraints applied followed by 1000 cycles with no constraints applied to further relax the system. The entire system was then gradually heated with the Langevin dynamics to 300 K while keeping the volume of the box fixed^55,56^. After properly thermalizing the systems to 300 K, the entire system was equilibrated for 100 picoseconds (ps) with 1 atm in NPT ensemble. However, for a production run the unbounded interface cut-off radius of 8.0 Å was set by using the Berendsen algorithm in NPT ensemble^57^. CPPTRAJ, a package of the AMBER program^58^ was used to evaluate system simulation trajectories whereas for analyzing and visualization of trajectories of MD simulations after every production run, the Visual Molecular Dynamics (VMD) was employed^59^.

### Binding energy calculations

To compute the binding free energies, Poisson–Boltzmann or Generalized Born and Surface Area Continuum Solvation (MM/PBSA and MM/GBSA) methods were used^60,61^. These methods sum up solvation-free energy G_solv_, gas-phase energy G_gas_, the van der Waals energies, and electrostatic interactions between free energies of the protein and the ligand using the total binding energy equation given below:

$$ \Delta {\text{G}}_{{{\text{bind}}}} = {\text{ G}}_{{{\text{complex}}}} {-} \, \left[ {{\text{G}}_{{{\text{receptor}}}} + {\text{ G}}_{{{\text{ligand}}}} } \right] $$

### Principle component analysis

Principal component analysis (PCA) is a method used for investigating protein structural changes and movement along the subspace during MD simulations. PCA takes the trajectory from MD simulations and extracts the dominant modes in the motion of the molecule^62–64^. To create the configurational space, a Cartesian coordinate system is used to generate a covariance matrix. By diagonalizing this matrix, a set of eigenvectors is obtained, which provides a vectorial description of each component of the motion by indicating its direction. Each eigenvector corresponds to a particular energetic contribution to the motion, represented by an eigenvalue. When the trajectory is projected onto a specific eigenvector, it highlights the time-dependent motion of the components in that vibrational mode. The time-averaged projection shows how the components of atomic vibrations contribute to this concerted motion mode^63^. PCA was performed on two sets of atoms: (i) all atoms of model 1 and 2 of the protein and (ii) only the backbone atoms of model 1 and 2. MD trajectories of the corresponding atoms were obtained using the CPTRAJ program^58^, and the analysis was conducted on 5000 frames in total extracted every 50 ns. PCA was run on all VoCs and their resulting MD trajectories.

## Results

### Protein thermodynamics and mutational effects

**Figure 1:**
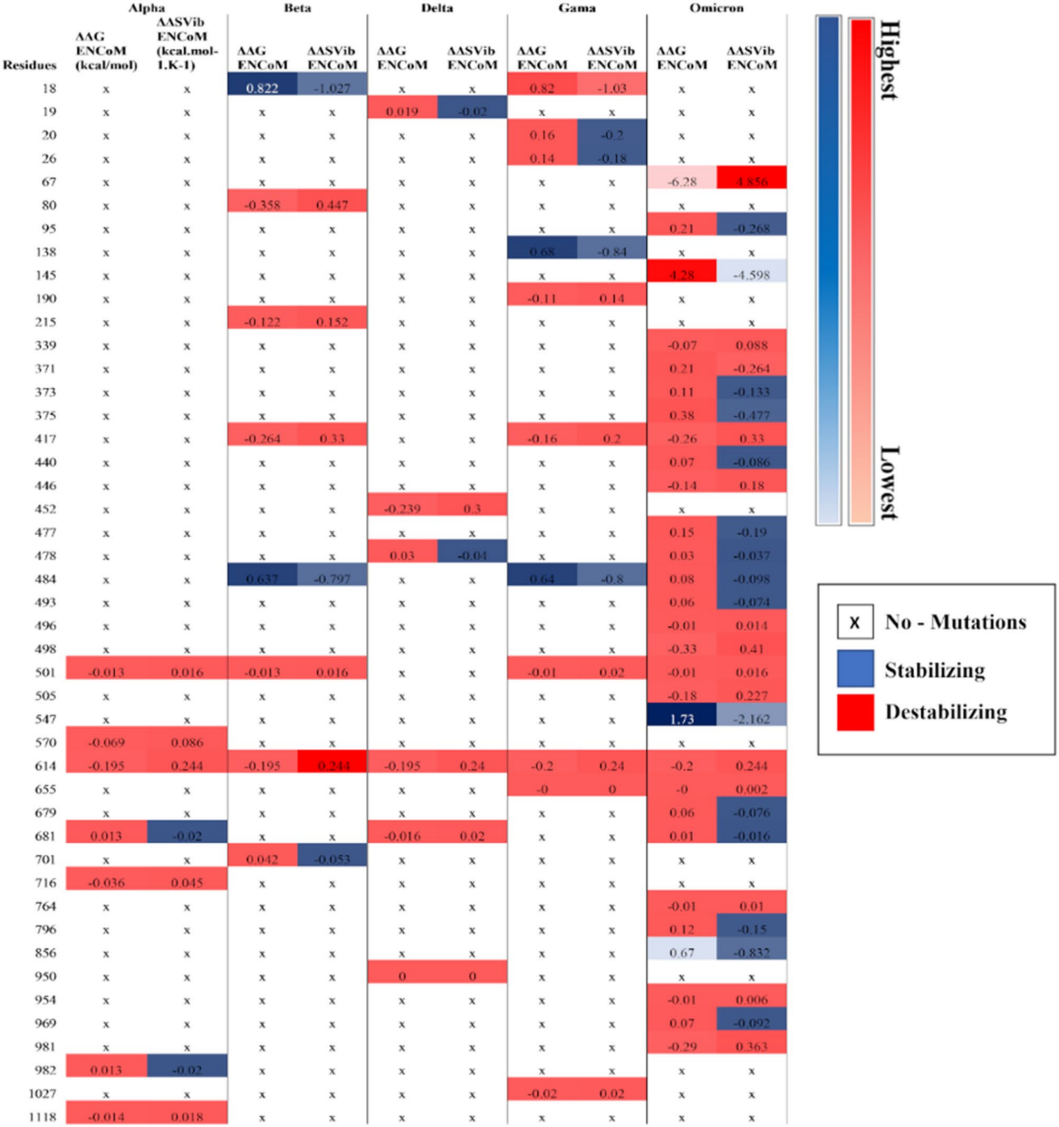
Normal mode analysis (NMA) of Dynamut demonstrating comparative thermodynamic effect of mutations on stability and flexibility of spike protein of all VoCs. Differences in free energy (ΔΔG) and variations in vibrational entropy (ΔΔS_Vib_ ENCoM) between the WT and other VoCs are mentioned from lowest to highest.

Based on the results of NMA, we predicted comparative thermodynamic effect of mutations on stability and flexibility of spike protein of SARS-CoV-2. To get insights into the effect of mutations on structural dynamics of VoCs under consideration, DynaMut calculated differences in free energy (ΔΔG) and variations in vibrational entropy (ΔΔS_Vib_ ENCoM) between the WT and VoCs presented in Fig. 1. It was observed that 22 substitutions; namely D614G, P681R, N501Y, K417N, L452R, D950N, Q954H, Q498R, G446S, G339D, L981F, N764K, Y505H, A67V, G496S, D215G, D1118H, T7161, A570D, H655Y, R190S, and T1027I resulted in dynamic behavior with increasing ΔΔG values that resulted in overall protein flexibility. Whereas NMA results revealed 6 substitutions: namely N856K, Y145D, T547K, L18F, D80A, and D138Y as stabilizing leading to the increase in rigidity in protein structure. Interestingly, another 17 substitutions; namely N679K, N440K, S375F, T478K, E484A, D796Y, S373P, T95I, Q493R, P681H, S477N, S371L, N969K, A701V, S982A, T20N, and P26S revealed themselves as destabilizing but caused an overall decrease in flexibility of the protein.

Out of these substitutions, there are 15 mutations reported in RBD of Omicron including 5 shared mutations mentioned in Table 1. While discussing shared mutations lying at RBD, substitution at K417N in all other variants except Gamma that substituted Asn417 with Thr exhibited comparatively higher ΔΔG ENCoM value of −0.159 kcal/mol than −0.264 kcal/mol imparting flexibility to the structure. This could be one of the foremost reasons that both K417N and K417T are associated with a decrease in ACE2 binding affinity in experimental studies^16^ and alternations at K417 tend to effect the binding affinity of class I and II antibodies at the RBD^65^. Whereas, the substitution at E484A exhibited unstable values with ΔΔG ENCoM 0.078 kcal/mol in Omicron compared to variants Beta and Gamma that exhibited stability with ΔΔG ENCoM values 0.637 kcal/mol. Notably, variations in vibrational entropy are substantial in defining the role of this immunodominant residue, which facilitates escape of antibody identified in different lineages of SARS-CoV-2^17,66^. Moreover, T478K that evolved in variants Omicron and Delta revealed itself as destabilizing but led to the increased rigidity. Due to the presence of T478K at RBM, a less accessible region of RBD, these residues that tend to make interactions in open conformational state of spike protein deliver compactness that can impact antigenicity and therefore hold precarious position while analyzing the effect of mutations on protein structure^10,67^.

The current study has also predicted thermodynamic effect of unique mutations present in the Delta variant such as L452R lying at RBD that led to the increased flexibility with ΔΔG ENCoM value 0.299 kcal/mol. Multiple studies have reported the evolution of this residue as a direct result of viral adaptation in response to a multitude of immunity^10,68^. It is a noteworthy substitution holding capability to neutralize multiple antibodies thus prompting the need to study the impact of these phenotypic changes in detail. Two more substitutions from Delta D950N and T19R exhibited maximum structural changes in turn leading to increased flexibility. Omicron on the other hand exhibited many new substitutions. We categorized it into four clusters based on their locations. Cluster I refer to substitutions S371L, S373P, and S375F, which contributed to a decrease in protein flexibility altogether. Cluster II refers to Q493R, G496S, both increasing protein flexibility followed by Q498R, which counteracted the effect of the previous two mutations by decreasing the vibrational entropy. Cluster III consists of N856K, N969K, T547K, and D796Y, which contributed to decreased flexibility according to Dynamut results. Cluster IV, however, has G339D, N440K, G446S, and Y505H substitutions, which are the most significant ones, leading to an increase in vibrational entropy except N440K. These results are in coherence with literature that associates residues of clusters I, II, and IV with improved stability whereas; cluster III is linked to weak fusogenicity^9^. Results yielded shed light on the combined and independent effects of these substitutions lying at RBD on thermodynamics of protein. Lastly, the conserved residues involved in cleavage of spike protein, such as P681H in Alpha and Omicron that existed in Delta as P681R exhibited contradicting results. P681H exhibited stabilizing values of ΔΔG ENCoM that is 0.013 kcal/mol whereas P681R exhibited destabilizing values of ΔΔG ENCoM −0.016 kcal/mol. The significance of mutation 681 which lies at furin cleavage site cannot be denied due to its presence with the neighboring residues D614G^20^. Both these substitutions have been regarded as critical in defining the rate of infection in different lineages^69^. Moreover, it has been observed that ΔΔG values that refer to the structural dynamics of protein can predict the binding equilibrium of interacting partners of SARS-CoV-2; namely S1 and S2 subunits.

### Mutations-induced intra/intermolecular changes in solution

**Figure 2:**
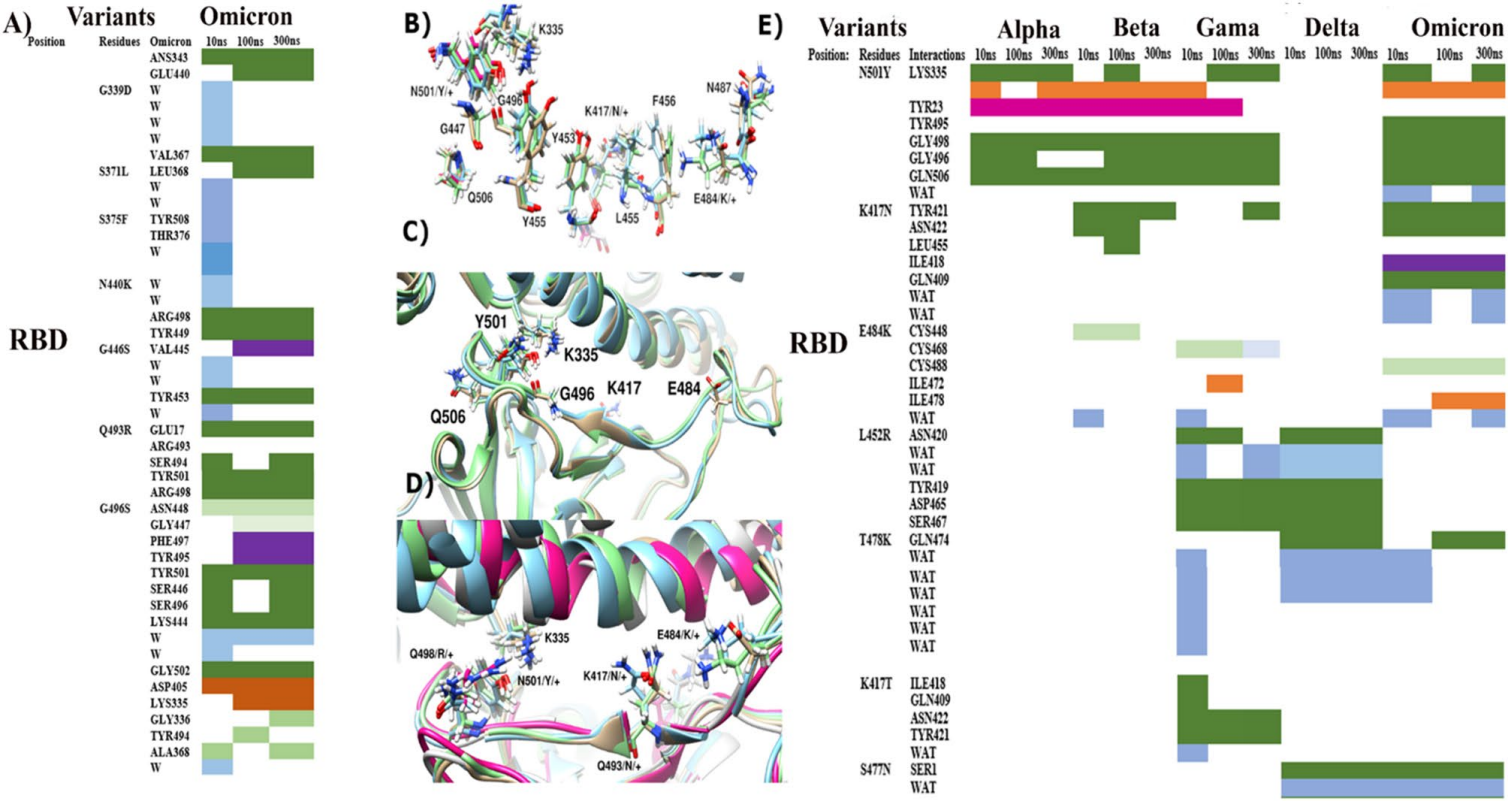
(**a**) Intermolecular interactions of recently emerged mutations in Omicron RBD with neighboring residues and solvent during 300 ns MD simulations (**b**) Homologous and presumably constrained interacting residues among all VoCs exhibit significant interactions with induced mutations. (+/−) signifies the charge. (**c**) Recently evolved mutations from cluster II reveal multiple interactions with neighboring residues (**d**) Highly diverse group of residues prone to variations in all SARS-CoV-2 sub lineages exhibit the capability to dramatically affect binding affinity. (**e**) Intermolecular interactions of shared mutations lying at RBD of all VoCs with neighboring residues and solvent during 300 ns MD simulations where (WAT) represents interactions with water molecules.

To further have insights into the effect of each mutation on intermolecular interactions with neighboring residues and solvent, MD simulations were carried out on trimers, monomers and RBDs of VoCs alone. Root mean square deviation (RMSD) of RBDs, monomers, and trimers based on 2800 ns MD simulations in total are demonstrated in supplementary Figs. S1, S2, and S3 respectively. MD simulations yielded interesting findings which are discussed here in detail. While discussing mutations at the RBD site (Fig. 2A–E), the most significant substitution at N501Y exhibited the maximum number of hydrogen bonds with neighboring residues till 300 ns namely, Lys335, Gly496, Gly498, and Gln506 with an additional hydrogen bond seen in Omicron with Tyr495. Similarly, K417N also exhibited maximum hydrogen bonds in Omicron variant with residues Tyr421, Asn422, and Gln409 while sustaining a hydrogen bond with Ile418 till 300 ns. Comparatively, substitution K417T in Gamma appears approx. onefold less collaborating while displaying 2 hydrogen bonds with Tyr421 and Asn422. Moreover, charged substitutions E484K and T478K exhibited a higher number of interactions with water molecules and neighboring cysteine residues namely Cys448, Cys468, and Cys488 in Beta, Gamma and Omicron depicted in Fig. 2E. Compared to other variants, Delta substitution at L452R exhibited strong hydrogen bonding with residues Tyr419, Asn420, Asp465, and Ser467 till 300 ns, depicting enhanced binding affinity with the ACE2 receptor. Notably, the remaining substitutions in Omicron exhibited a higher number of bonds with water residues while only S477N, Q498R, and Y505H sustained it till 300 ns of MD simulations depicted in Fig. 2A. All hydrogen bonds with their time series and percentage of occupancy during MD simulations are listed in supplementary file Figs. S4–S8.

Furthermore, newly acquired substitutions in clusters I and II of Omicron primarily G496S and Q498R revealed a twofold increase in interactions. These enhanced integrating moieties observed in Omicron are predominantly due to hydrophobic and hydrophilic contacts, particularly at the RBD-up region including Q493R, G496S, Q498R, N501Y, and Y505H followed by S371L, S373P, and S375F. A higher number of positively charged residues present at the interface of Omicron is displayed in Fig. 2E compared to other variants while harboring a loss of K417N that participated significantly in establishing interactions in the Delta variant.

Lastly, the previous studies that reported the significance of distal region furin site and D614G^20^ are in coherence with the current analysis. Strong hydrogen bonding with Ala647 in both Delta and Omicron variants is observed whereas, P681R in Delta expressed comparatively lesser water-mediated interactions as compared to P681H in Omicron, which exhibited threefold stronger interactions with water. These water-mediated interactions may provide one of the reasons for the enhanced stability of Omicron compared to Delta as reported in multiple studies^11,70^. In this context, other significant substitutions at furin site and NTD were also analyzed. N-terminal substitution in Delta namely T19R displayed strong hydrogen bonding with ACE2 receptor namely, Asp251, Ser253, Ser254, and Trp256 compared to Omicron N-terminal substitutions A67V, T95I, and Y145D that revealed a higher number of non-covalent interactions in this region. Although Delta and Omicron both impart stronger contacts in this region as depicted in supplementary Fig. S9, Omicron is observed participating more actively to hold the S1 and S2 subunits together before splitting apart as a result of cleavage and subsequent release into the cell^8,71^. Furthermore, Omicron also holds an additional 3-residue insertion at position 214 (ins214EPE) at the furin site that exhibited strong hydrogen bonding with Asp788, Gln793, and Gly794. However, the role of ins214EPE in Omicron needs more corroboration, which is discussed in detail in forthcoming sections.

### Molecular determinants responsible for relative binding affinities

**Figure 3:**
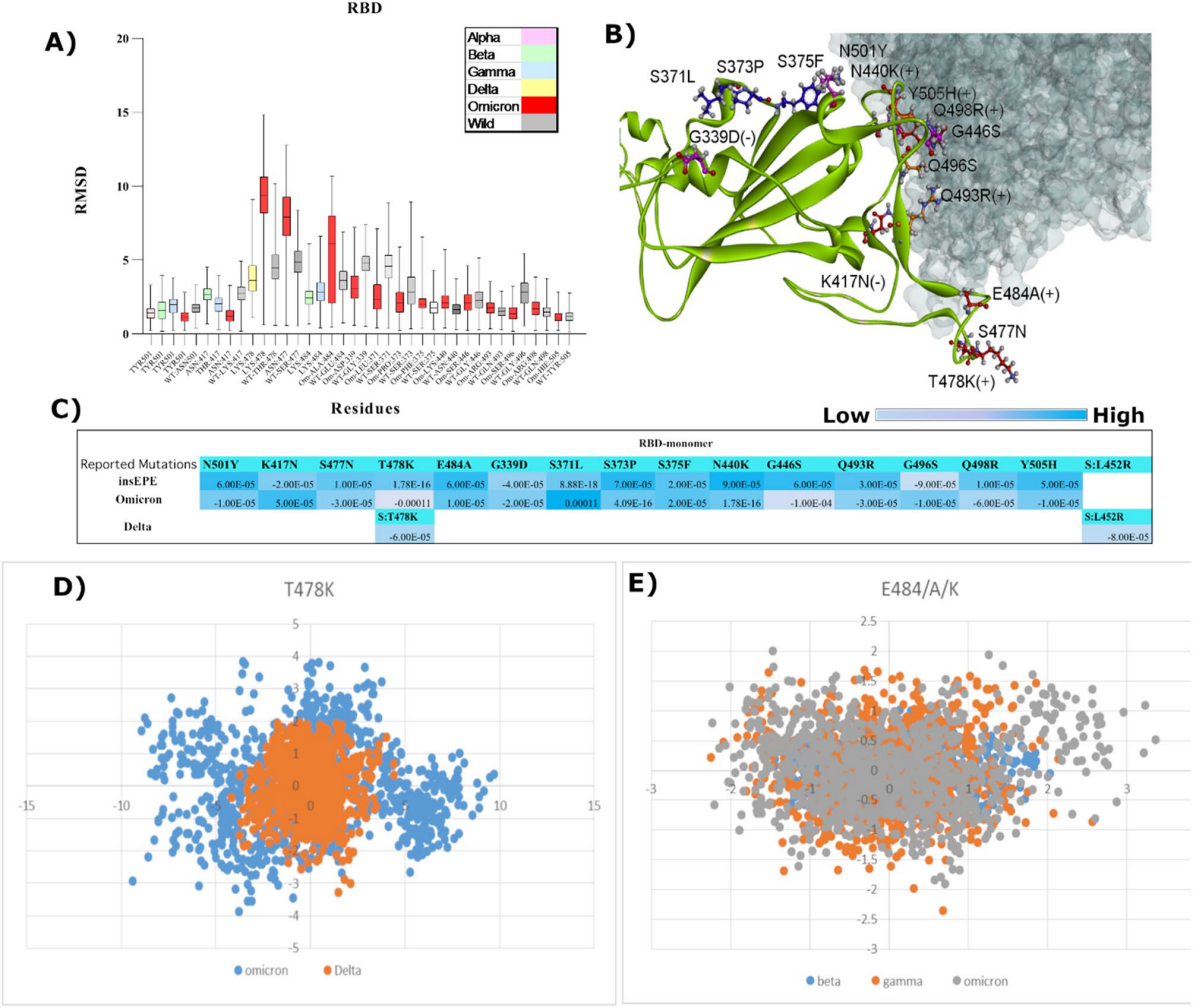
(**a**) RMSD of significant residues lying at RBD of all VoCs and WT are represented by box plots. (**b**) Snapshot of spike protein (green ribbon) exhibit positions of mutated residues lying at RBD which are directly involved in making interactions with ACE2 receptor (grey surface) (**c**) Comparative free binding energy profile of reported mutations lying at RBD (**d**) Comparative PCA of Delta and Omicron mutation T478K exhibit residual distribution along the subspace (**e**) Comparative PCA of Gamma, Delta and Omicron mutation E484/A/K exhibit residual distribution along the subspace.

**Figure 4:**
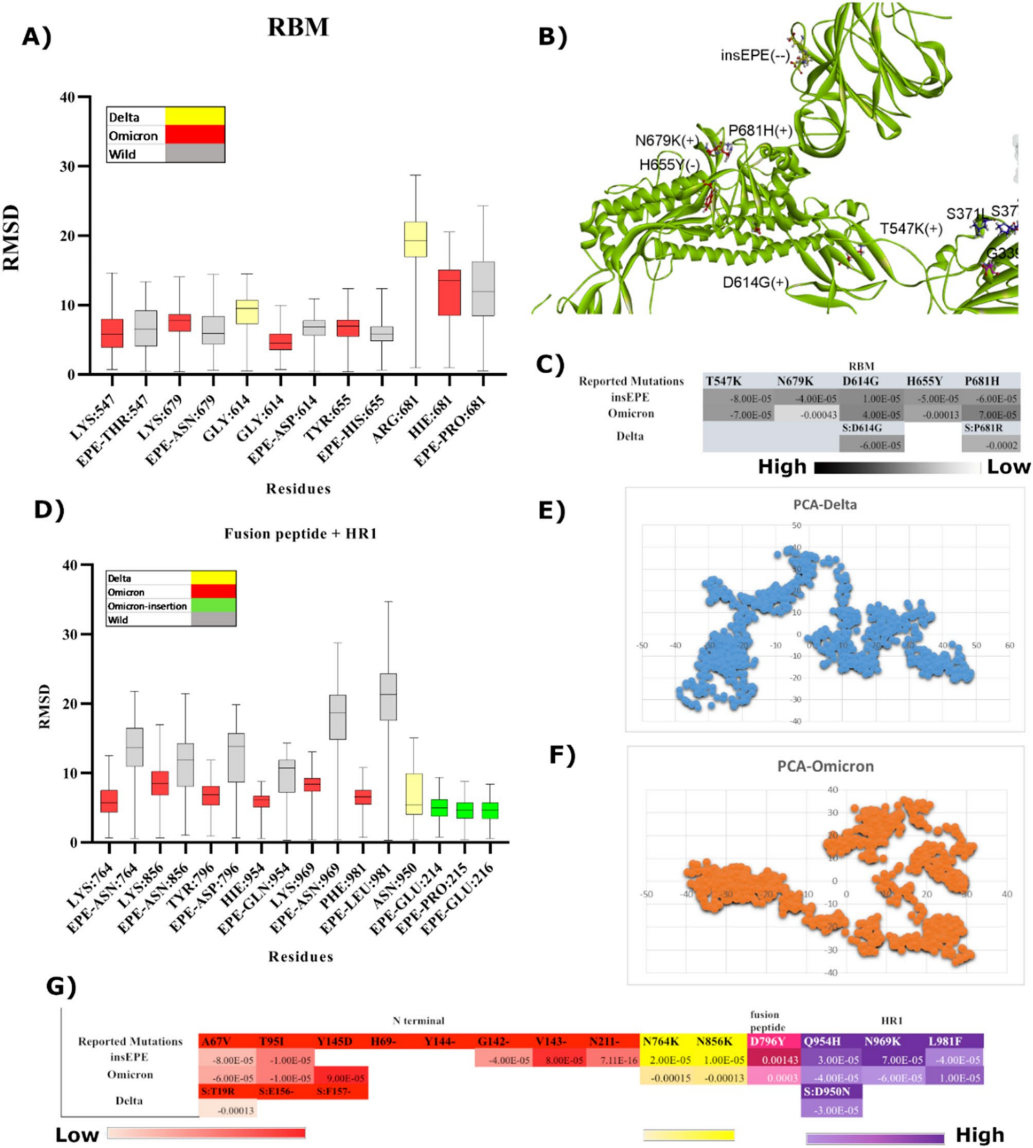
(**a**) RMSD box plots of RBM of Delta, Omicron, and WT monomers (**b**) Snapshot of spike protein (green ribbon) exhibit the position of mutated residues at RBM critical for compact conformation of Omicron (**c**) Comparative free binding energy profile of reported mutations at RBM (**d**) Comparative RMSD box plots of mutated residues at fusion peptide site and HR1 along with insertion EPE lying at position 214 (**e**) PCA of monomer Delta exploit different energy minima while sampling trajectories (**f**) PCA of monomer Omicron sample in completely opposite direction compared to Delta monomer. (**g**) Comparative free binding energy profile of reported mutations at N-terminal, fusion peptide site, and HR1.

RBD region of spike protein that directly interacts with ACE2 receptor carries the maximum number of mutations thus playing an important role in viral transmissibility and adaptability^72^. The increased number of mutations at RBD of Omicron is certainly correlated with its ability to facilitate cell entry^24^. We calculated RMSD and structural changes of reported mutations along with binding affinities of RBD of all VoCs depicted in Fig. 3A–E. Substitutions exhibiting significant changes were then compared by performing PCA to assess their residual structural contribution. Delta RBD exhibited fivefold increased binding affinity than WT and 2.8-fold increased affinity than Omicron RBD as depicted in Fig. 3C. Results comprising isolated RBD of all VoCs attached with ACE2 receptor exhibited the highest RMSD for Omicron residues Asn477, Lys478, and Arg484 → Ala compared to Arg484 → Lys in Alpha and Gamma as depicted in Fig. 3A. These two residues, Arg484 and Lys478 both added a positive charge and exhibited different mobility for Omicron compared to other VoCs when PC1 and PC2, the projections of essential subspace were plotted (Fig. 3D,E). Whereas Omicron Tyr501 and Asn417 exhibited the lowest RMSD compared to other VoCs but higher than its WT Asn501 and Lys417. Both residues exhibited similar cluster distribution although K417N added a negative charge while N501Y added a positive charge (Fig. 3B). Overall cluster projection of RBDs of all VoCs is depicted in Fig. S10 of the supplementary file. Furthermore, the overall cluster projection of monomers including all regions RBM, fusion peptide and HR1 demonstrated in Fig. 4A–G revealed that Omicron and Alpha exhibited similar conformer distribution along the subspace whereas Delta, Gamma, and Beta sampled in opposite directions (Fig. 4E,F).

Apart from shared mutations, newly acquired mutations in Omicron cluster I exhibited lower RMSD and robust binding affinity values when compared to WT (Fig. 4A,G). Similarly, cluster III was observed displaying low RMSD and higher binding affinity signifying a more compact domain organization with the ability to impart positive charges as displayed in Fig. 4B. Cluster II which is directly involved in ACE2 binding exhibited comparatively higher RMSD values but the same binding affinity pattern was observed with two positive charged residues. Moreover, residues including P681H/R and D614G, which are both positively charged along with ins214EPE hold a significant standpoint with the capability to impart two negative charges (Fig. 4B). However, lower RMSD values and weak binding affinity for ins214EPE were observed compared to WT (Fig. 4D). Comparative details of binding energies are given in Table S1 of the supplementary file. To conclude, the overall combined effect of Omicron substitutions signifies the importance of charge distribution in defining the capability to reinforce stability due to a higher number of mutations into polar residues.

### Conformational rearrangements underpin the active conformation

**Figure 5:**
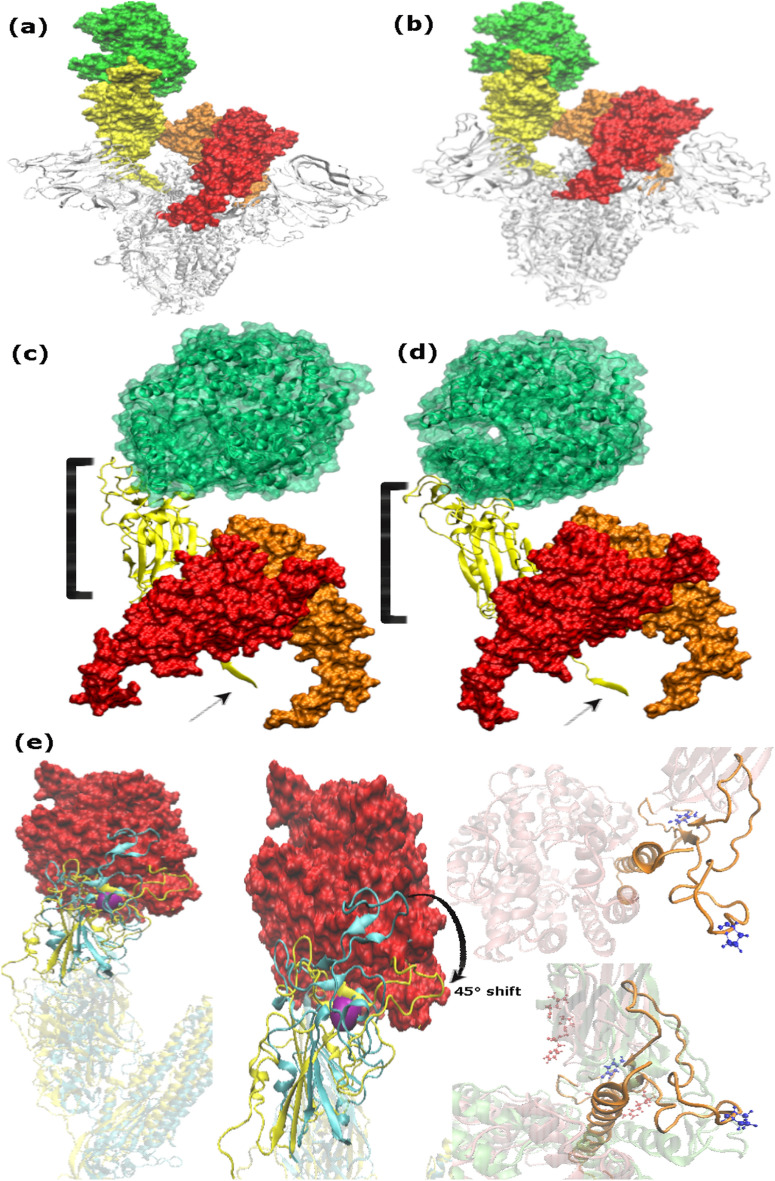
Conformational changes observed in different variants of spike protein during a simulation run. (**a**) WT trimer and (**b**) Omicron trimer, highlighting receptor in green and the three RBD domains in orange, red, and yellow. The ‘Up’ and ‘Down’ conformation of RBD is visible while interacting with the receptor and during no interaction, respectively. (**c**) and (**d**) zoomed-in view of the Delta and Omicron monomer respectively to display difference in structural compactness and stability. (**e**) Comparison of structural and conformational changes that occur during the binding of Delta and Omicron variants to ACE2 receptor. Zoomed in view of superimposed RBD domain and HR1 peptide where purple surface depicts L452R.

**Figure 6:**
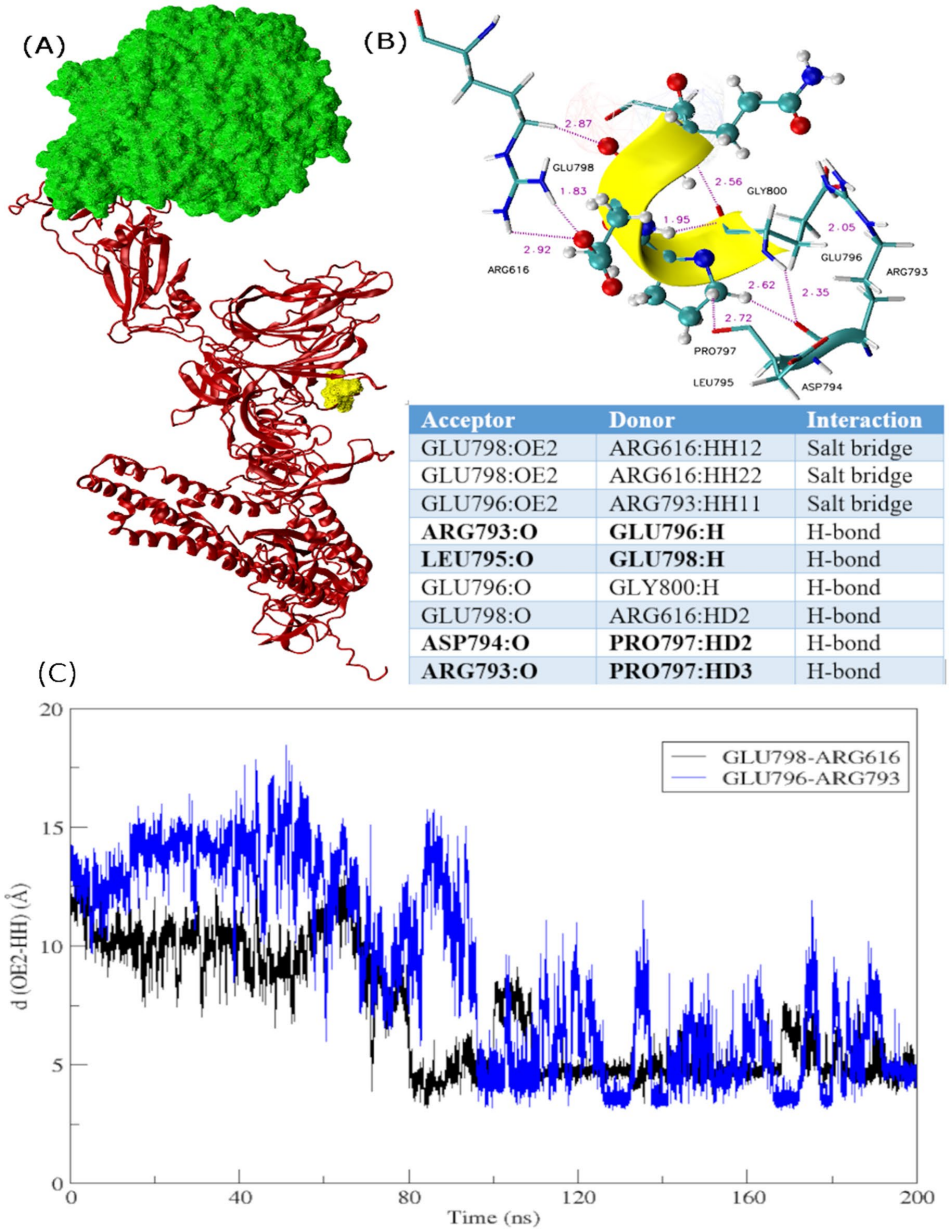
Intermolecular interactions surrounding the ins214EPE in the WT spike protein after 200 ns of the simulation run. (**a**) ACE2 receptor is in surf representation in green whereas the rest of the monomer is in ribbon representation (**b**) Zoomed in view of ins214EPE and interacting protein residues forming salt bridge and hydrogen bond interactions (**c**) Assessing the distribution of distances between residues involved in making salt bridges during MD simulations.

**Figure 7:**
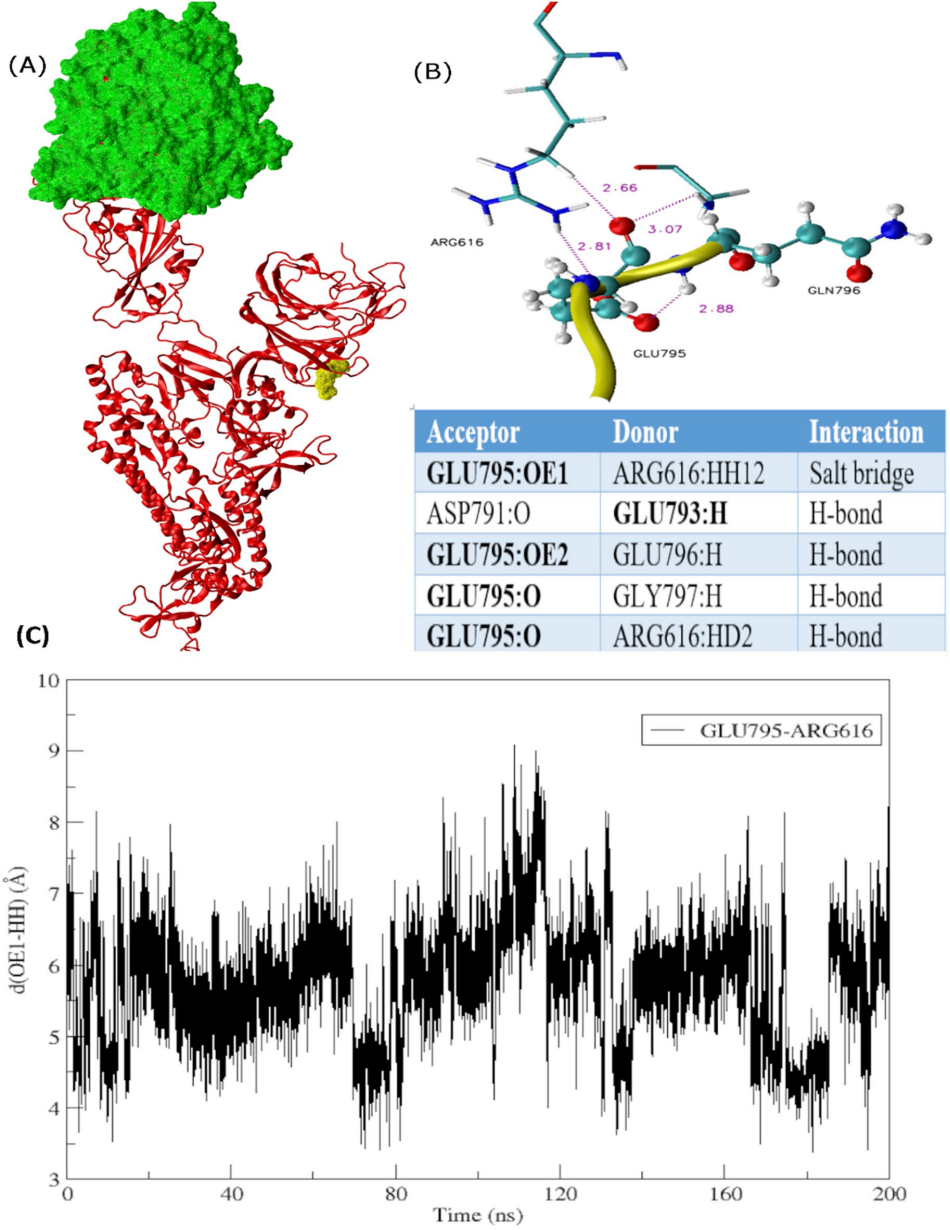
Intermolecular interactions surrounding the ins214EPE in the Omicron spike protein after 200 ns of the simulation run. (**a**) ACE2 receptor is in surf representation in green whereas the rest of the monomer is in ribbon representation (**b**) Zoomed in view of ins214EPE and interacting protein residues forming salt bridge and hydrogen bond interactions (**c**) Assessing the distribution of distances between residues involved in making salt bridges during MD simulations.

Furthermore, to draw inferences about comparative structural and conformational rearrangements, analysis of monomers and trimers was carried out. It was observed that the up-RBD remains in the up-configuration while binding to the receptor whereas the down-RBD with no receptor attached exhibited a down-configuration (Fig. 5A,B). Similar findings were observed while comparing the monomer Omicron with Delta depicted in Fig. 5C,D. 45-degree conformational shift in ACE2 attachment site in Delta was observed, primarily due to the substitution L452R exhibited in zoomed-in view in Fig. 5E. Consequently, the architecture of up-RBD region deviated leading to a clockwise rotation, which subsequently drifted down-RBD farther apart. Whereas much more compact organization was observed in Omicron intersubunits with the addition of S371L, S373P, S375F, and Q493R, G496S, Q498R, and N501Y substitutions (Fig. 5E), which seem to be the facilitator of the active conformational state. Moreover, interactions between the ACE2 receptor and RBD domain remained more widespread in Omicron compared to Delta and WT. The surface area of receptor interacting with Omicron RBD had intensive hydrogen bond networks along with a salt bridge formed between Glu798 and Arg616 depicted in Fig. 6. This interaction plays an important role in keeping the receptor compact and more interactive with Omicron RBD throughout the simulation run. Additionally, Fig. 7 exhibits another salt bridge formed between Glu795 and Arg616 of NTD triggering more stable conformational changes in Omicron.

### Role of EPE insertion in Omicron

**Figure 8:**
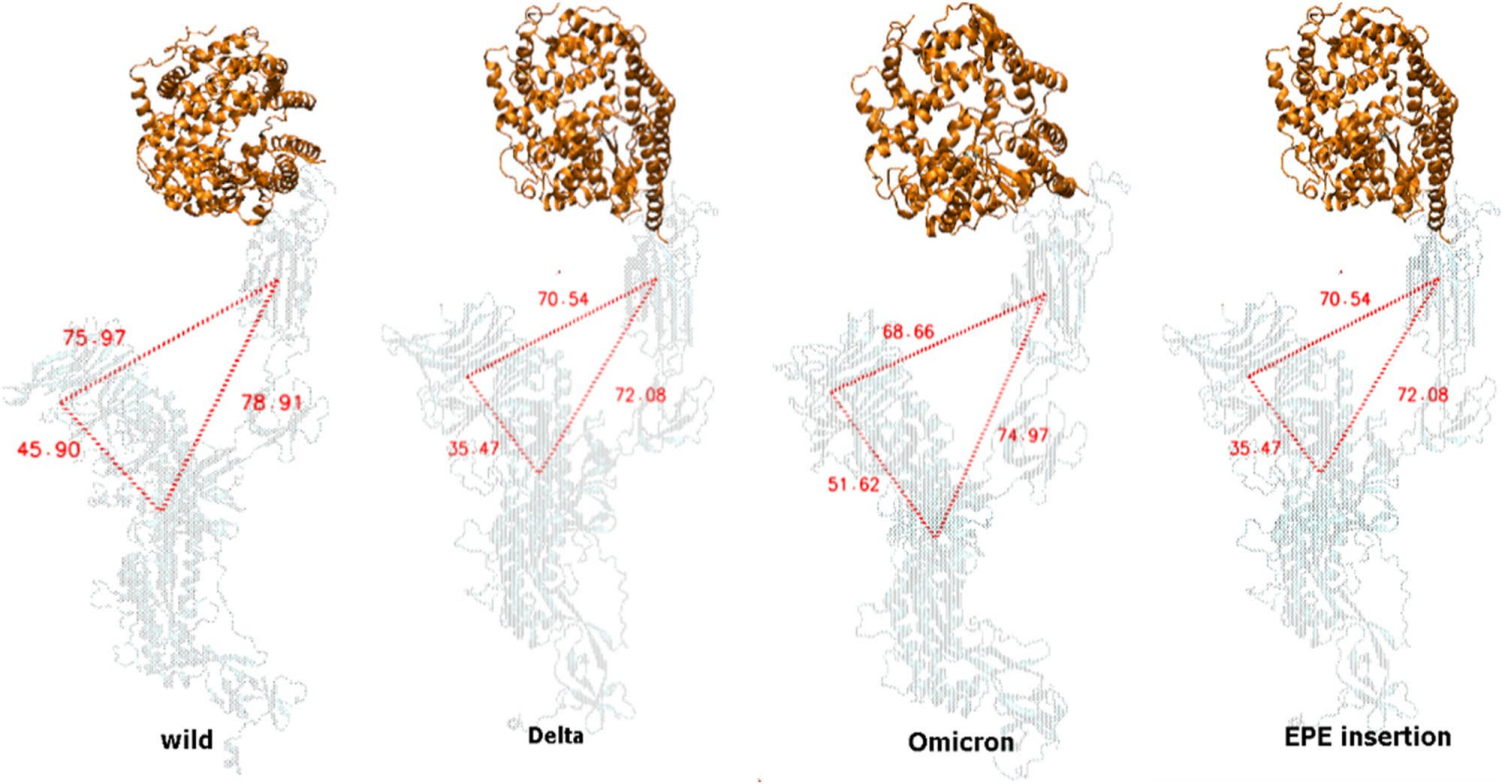
Monomers at 200 ns simulation run each. Insets representing the diverse structural shifts and altered angles formed (RBD-HR1-NTD) in the variants that were triggered by ACE2 binding. Orange ribbon displays the receptor.

Multiple insertions in protein evolution over time play an important role in surfacing novel protein architectures^73^. The number of insertions and deletions within the protein sequence is directly proportional to the evolutionary distance^65,73^. However, in the case of VoCs, Omicron has the highest number of substitutions, unique insertions, and deletions compared to other variants. Simulation results while exploring conformational stability and compactness of spike protein suggested that Omicron has a more stable structural orientation extensively interacting with the ACE2 receptor while forming improved intersubunit contacts. As an outcome, the ACE2 binding has caused notable and diverse structural shifts in different variants of spike protein. The RBD-HR1-NTD have exhibited varying distances and altered angles in all four complexes depicted in Fig. 8, which reflect the role of mutations in controlling structural orientations while keeping protein in an active conformational state. Findings demonstrate 69.29 Å, 73.20 Å, 62.52 Å, and 56.83 Å angular distances between RBD-HR1-NTD of WT, Delta, Omicron, and ins214EPE monomers crucial for viral stability of spike protein that is necessary for viral transmission and immunogenicity. These findings propose that ins214EPE was necessary to better characterize the process of substitution in Omicron. Simulation analysis of WT with ins214EPE exhibited salt bridge interactions with neighboring Arg residues shown in Figs. 6, 7. In addition to this, six potential hydrogen bonds were observed during a simulation run. Notably, the ins214EPE occupied mainly the loop regions of NTD and has not disturbed the folding of protein core structure while allowing the protein to explore its conformational space and develop novel substructures.

### Role of water-mediated interactions

Subsequent mutations of residues can increase or decrease the number of water molecules interacting with the core region of macromolecule resulting in gain or loss of protein stability^74^. In the current study, we have observed that water-mediated contacts kept on increasing and getting stronger with each upcoming Covid variant. For instance, nearly all mutated residues of Omicron exhibited formation and breaking of hydrogen bond interactions with water throughout the simulations. However, if we look at the simulation analysis of WT and early-stage variants of Covid such as Alpha, Beta, Gamma, and Delta, the interaction of water molecules with mutated residues can be seen as minimal or not at all. Water is an integral component of biomolecular systems that mediate hydrogen bonds and electrostatic interactions^75^. Significant amount of research data highlights the invaluable role of hydration forces in guiding the structure, stability, function, and dynamics of proteins^74,76^. Water regulates protein folding by defining hydrophobic interactions that glue hydrophobic residues stabilizing the folding process^76^. Water also interacts with protein backbone, surface residues, and sidechains while controlling the formation of hydrophobic cavities. Mutations of a notable number of hydrophobic residues into hydrophilic residues have resulted in increased water-mediated interactions in Omicron leading to a more compact structure and stable behavior of the protein compared to other VoCs. Increased affinity of Omicron towards ACE2 receptor and improved role of solvent-mediated interactions has influenced its biological functionality. Consequently, Omicron being highly stable, and transmissible but much less infectious has been favored in the process of natural selection.

## Discussion

Owing to the presence of isolated RBD, monomeric and trimeric Cryo-EM structures of spike protein in complex with ACE2, a comprehensive comparative mutational and structural analysis on Omicron and other VoCs elucidated significant structural changes, which may act as facilitators in explaining the higher risk of Omicron transmission rate and reveal interesting facts about its compromised viral fusion ability.

The effect of independent mutations on thermodynamics of spike protein in all five variants revealed dynamic behavior of conserved mutations such as K417/N/T, E484/A/L, N501Y, D614G, and P681R/H. ΔΔG values reveal that successive mutations at RBD including recently emerged Omicron clusters could be directly involved in the enhanced transmissibility of Omicron. Interestingly, mutation P681H in Omicron forged decreased NMA values compared to P681R in Delta that exhibited weak stability or loose attachment suggesting a direct link with enhanced replication and cleavage of S1 and S2 subunits of Delta as reported in previous studies^67^.

The above discussion is largely supported by in silico models and simulations carried out to disclose the molecular basis of improved stability of Omicron. We characterized amino acids involved in making significant interactions with RBM + RBD based on their binding potential in different groups. Homologous residues exhibiting high binding potential in establishing strong interactions among all VoCs are Lys335, Gly447, Gly498, Gly496, Gln506, Leu455, Tyr449, Tyr453, Asn487, and Phe456 depicted in Fig. 2B. Role of these conserved residues is presumably constrained due to their involvement in protein folding or ascertaining binding affinity for ACE2. Whereas other groups of residues prone to undergo mutations in all sublineages are G446, Y505, K417, E484, and cluster II mentioned in Fig. 2C,D. It is therefore deduced that residues that are characterized as molecular determinants participating in protein folding have comparatively less potential to mutate in upcoming variants of Omicron or Deltacron or any other variant. However, the role of mutations at NTD and furin site that are associated with an enhanced transmission rate of Delta and Omicron^77,78^, particularly P681R that emerged in Delta and mutated into P681H in Omicron demonstrated threefold increased electrostatic interactions with water and enhanced hydrogen bonding depicted in supplementary Fig. S2 and Fig. 4C. These findings signify sturdier molecular bridges holding the S1 and S2 subunits of Omicron together.

Further investigations on structural dynamics and conformational orientation during MD simulations reiterate compact intersubunits in Omicron compared to Delta which exhibited angular movements due to L452R depicted in Fig. 5E. Similar cluster projections between Alpha and Omicron compared to Delta and other VoCs exploited different energy minima while sampling trajectories. Another significant finding anticipated to support compact domain organization in Omicron is unique ins214EPE. MD simulations revealed structural shifts and significant differences in angular distances between RBD-HR1-NTD of WT, Delta, Omicron, and ins214EPE monomers demonstrated in Fig. 8. Ins214EPE is therefore crucial for viral stability of spike protein to understand the biological consequences of recently acquired mutations. The observed differences particularly between Omicron and Delta based on certain mutations directly affect protein stability and binding that shed light on possible reasons of divergence between transmission rate and fusogenecity of both the variants.

Current study likewise proposes a substantial role of hydration forces based on an influx of solvent-mediated interactions with each Covid variant. As far as Delta and other VoCs are concerned, no significant, solvent-mediated interactions give the impression. Thus, the interplay between protein and solvent is critical in shaping Omicron function and dynamics. Furthermore, Delta and Omicron expressed a higher number of mutations into charged residues + 4 and + 9 respectively compared to their successors Alpha, Beta, and Gamma. Role of hydrations forces and positively charged residues explained by Nie et al. in previous studies support the fact that an increased number of mutations in Omicron drastically affect the binding capability of antibodies to these cationic patches on the interface of spike protein^79^.

Based on these findings, it has been discussed that mutational spread is positively related to viral fitness, which has been defined in literature^80^. For instance, current research data suggests that Covid19 variants employing hydration forces along with other electrostatic interactions are more compact; thus, play a significant role in protein folding and function that are prevalent among the human population. The growing number of water mediated contacts observed in these variants underscores the influence of hydration forces on binding kinetics, affinity, and specificity of the receptor-ligand complex. Notably, literature supports the essential role of water in rendering the binding site accessible to both ligand and receptor^74–76^. Additionally, the involvement of water in enhancing the solubility of ligands, particularly preceding their interaction with the target protein is well documented. Water-mediated hydrogen bonds contribute to the stability of the complex and have the potential to induce conformational changes in the binding pocket structure. Crucially, hydration forces can impact entropy changes during ligand binding, thereby altering the thermodynamics of the interactions. To gain deeper insights into the hydration sites on the surface of Covid19 variants, future studies should leverage extended MD simulations, solvation free energy calculations, and thermodynamic analyses. These computational results can be further validated through experimental solubility data, providing meaningful insights for the improved design of drugs and therapeutics.

### Supplementary Information

Supplementary Information.

## Data Availability

A list of mutated sequences of all Covid variants was retrieved from the GISAID database (https://www.gisaid.org/hcov19-variants/). Mutations were carried out with DynaMut and three-dimensional (3-D) structure of WT and all variants of Covid19 were generated using SWISS-MODEL (https://swissmodel.expasy.org/). The Discovery studio visualizer is freely available after registration which provides limited functionality and is used for visualizing results retrieved in this study. Molecular Dynamics simulations and binding free energy calculations were performed using AMBER 16 suite. The AmberTools suite is free of charge, and its components are mostly released under the GNU General Public License (GPL).
