# Supplementary material for: Dynamics of water-mediated interaction effects on the stability and transmission of Omicron
Source: Sci Rep. 2023 Nov 28;13:20894. doi: 10.1038/s41598-023-48186-2 (PMC10684572; doi:10.1038/s41598-023-48186-2)
Supplement: Supplementary file 1 — Supplementary Information. [file 41598_2023_48186_MOESM1_ESM.pdf]

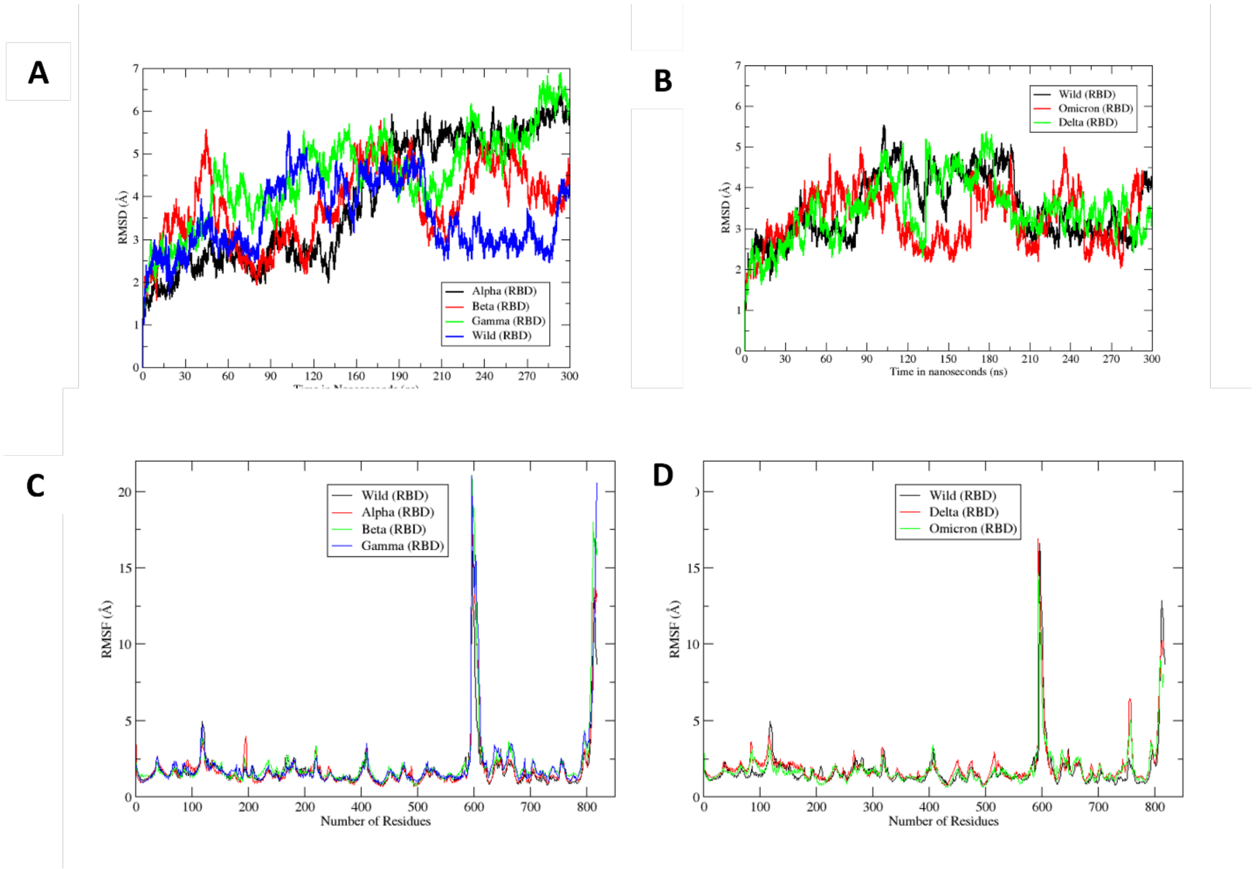

Figure S1: Comparative trajectories analysis of 300 ns MD simulations of SARS-CoV-2 RBD of VoCs. a) RMSD of Alpha, Beta, Gamma, and WT variants b) RMSD of Delta, Omicron, and WT variants c) RMSF of Alpha, Beta, Gamma, and WT variants d) RMSF of Delta, Omicron, and WT variants.

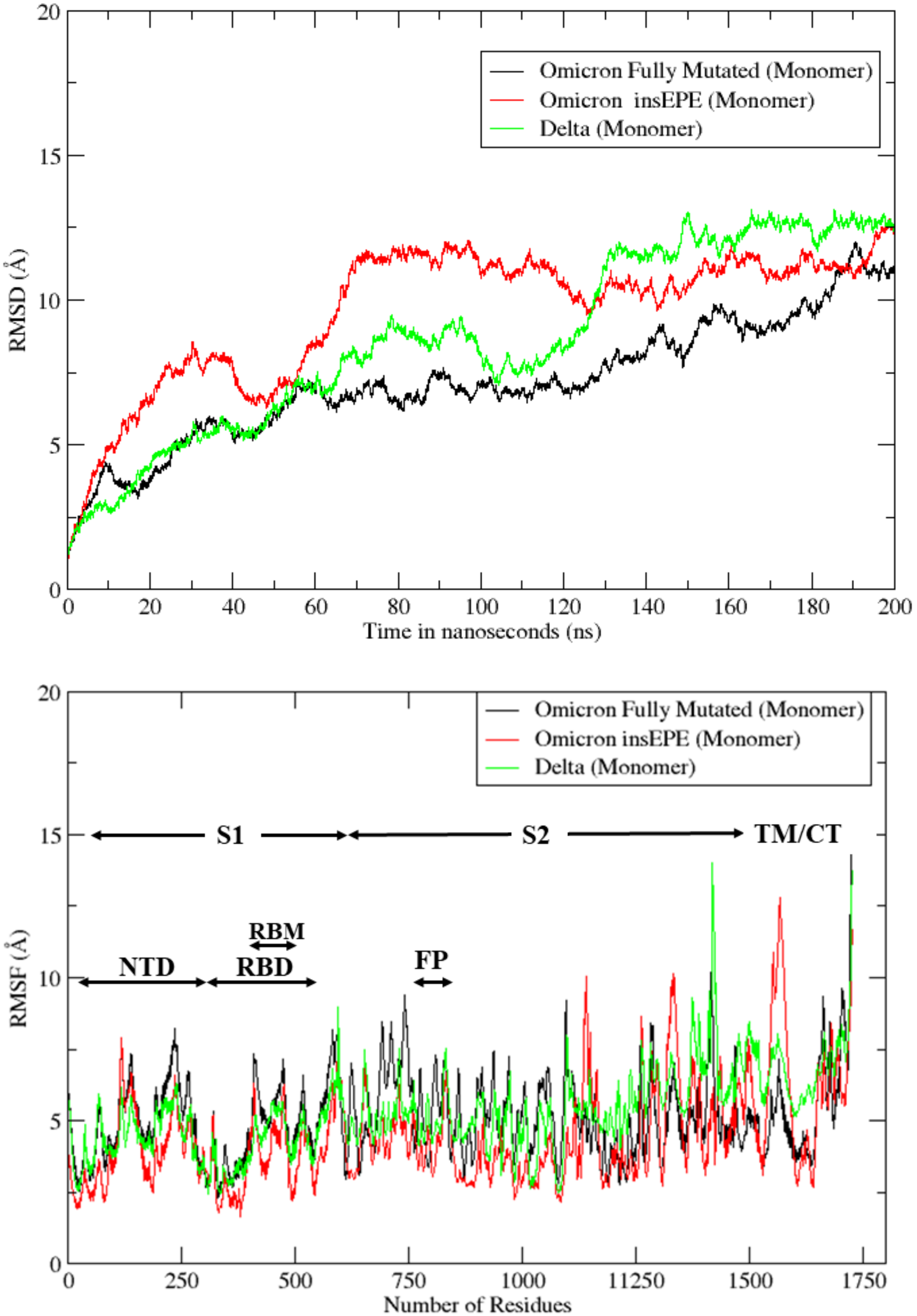

Figure S2: Trajectories analysis of 200 ns MD simulations of SARS-CoV-2 monomers. a) RMSD of Delta, Omicron, and WT variants b) RMSF of Delta, Omicron, and WT.

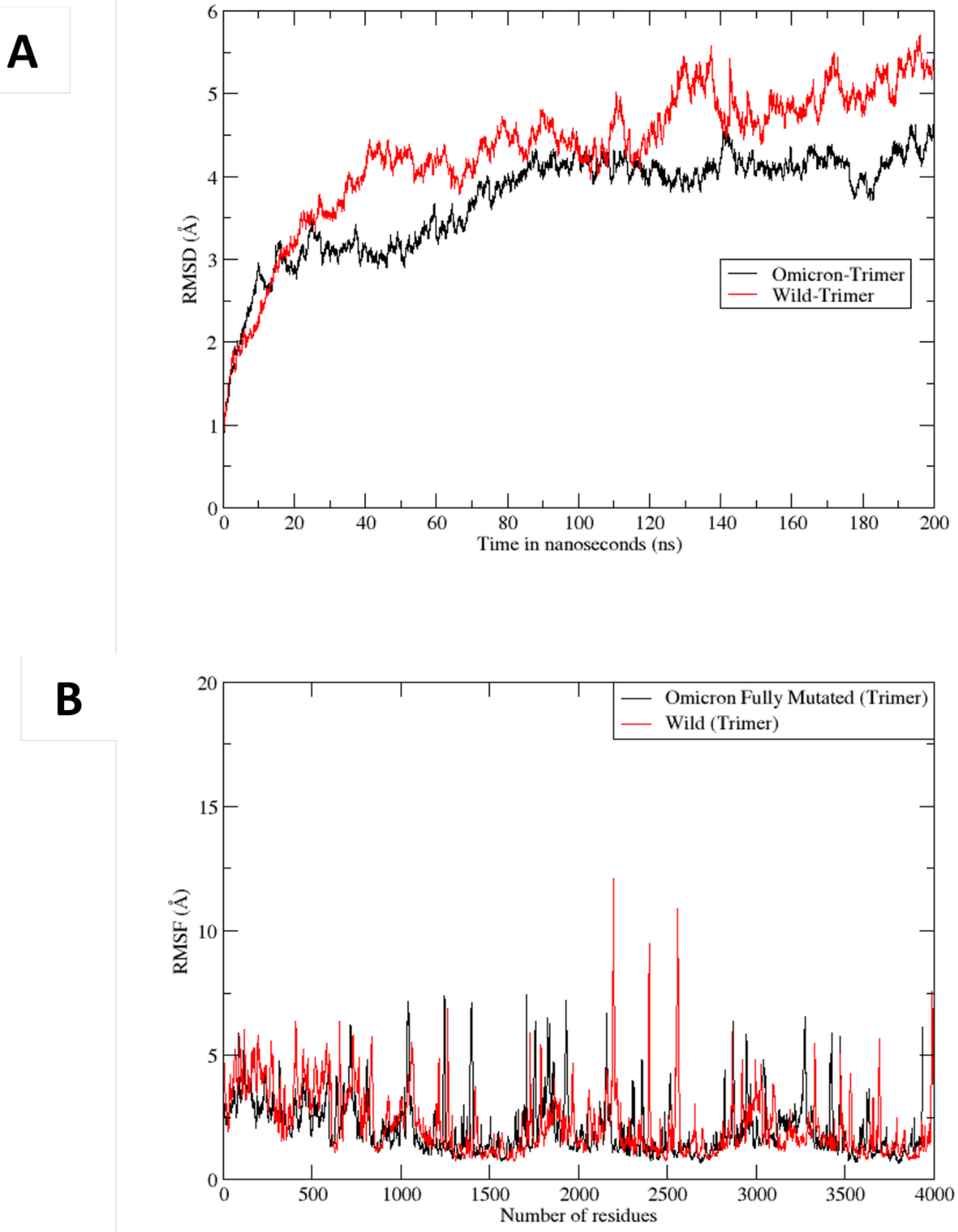

Figure S3: Trajectories analysis of 200 ns MD simulations of SARS-CoV-2 trimers. a) RMSD of WT and Omicron variants b) RMSF of WT and Omicron variants.

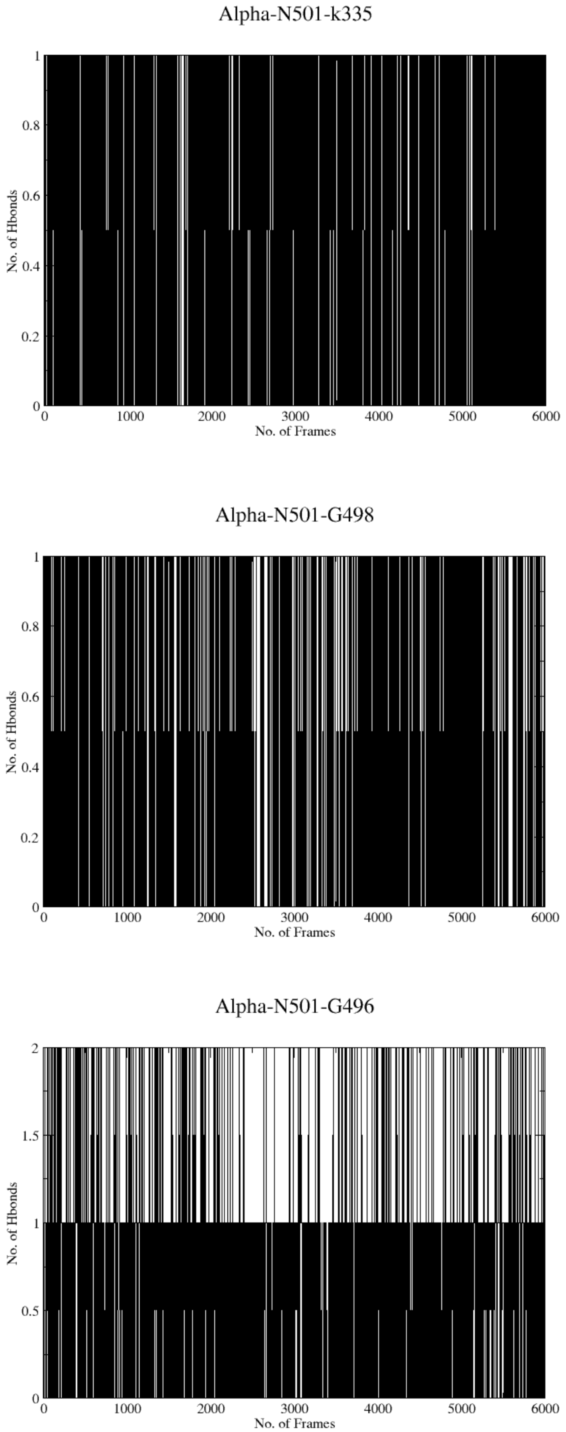

Figure S4: Hydrogen bond interactions observed during 300 ns MD simulations of RBD of variant Alpha.

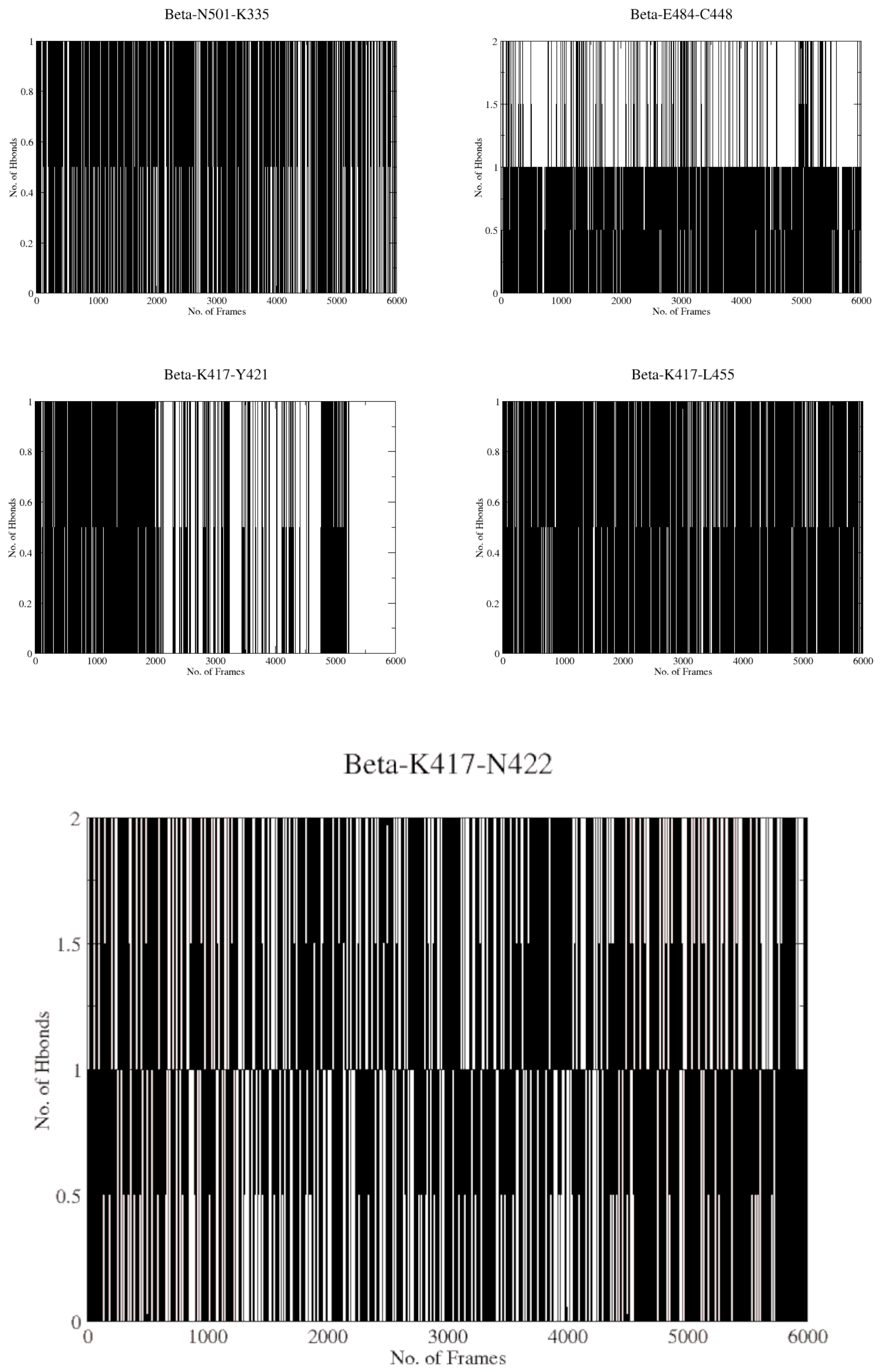

Figure S5: Hydrogen bond interactions observed during 300 ns MD simulations of RBD of variant Beta

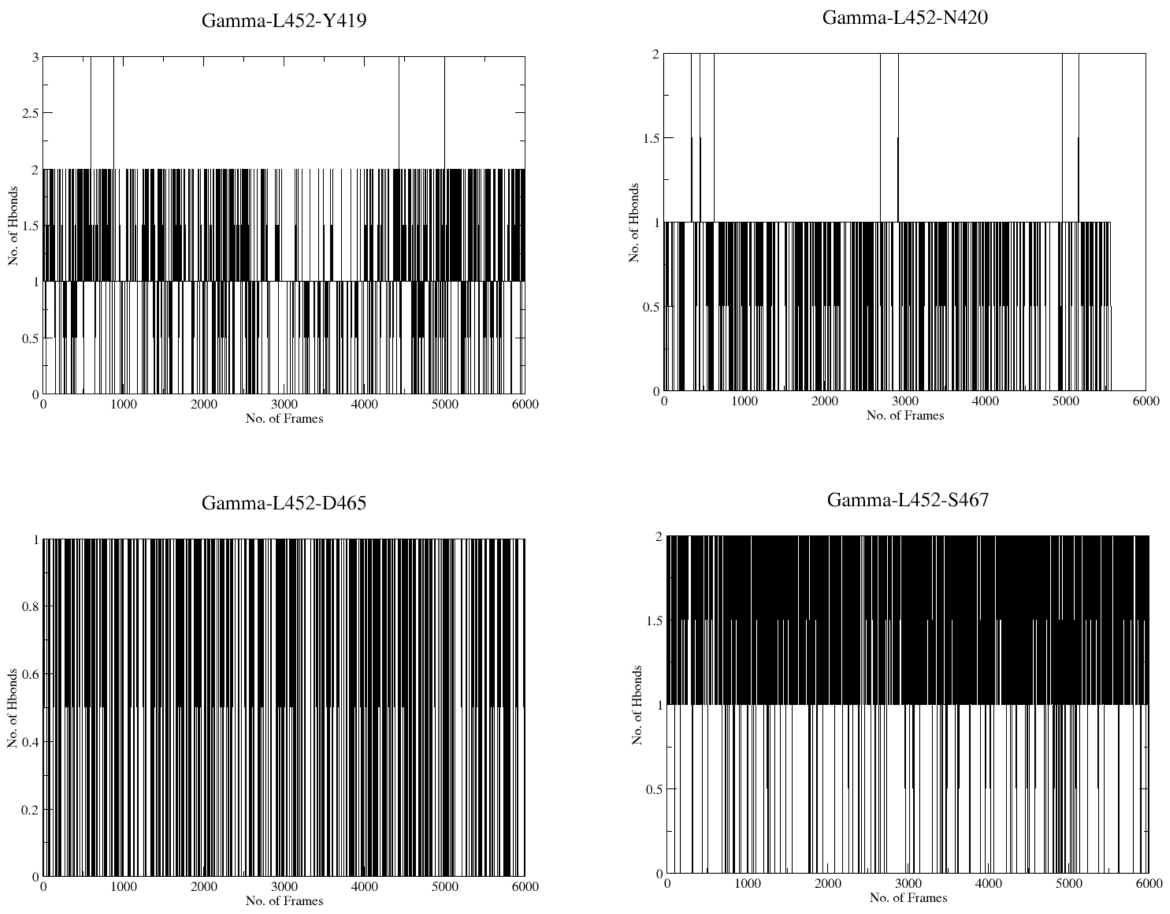

Figure S6: Hydrogen bond interactions observed during 300 ns MD simulations of RBD of variant Gamma.

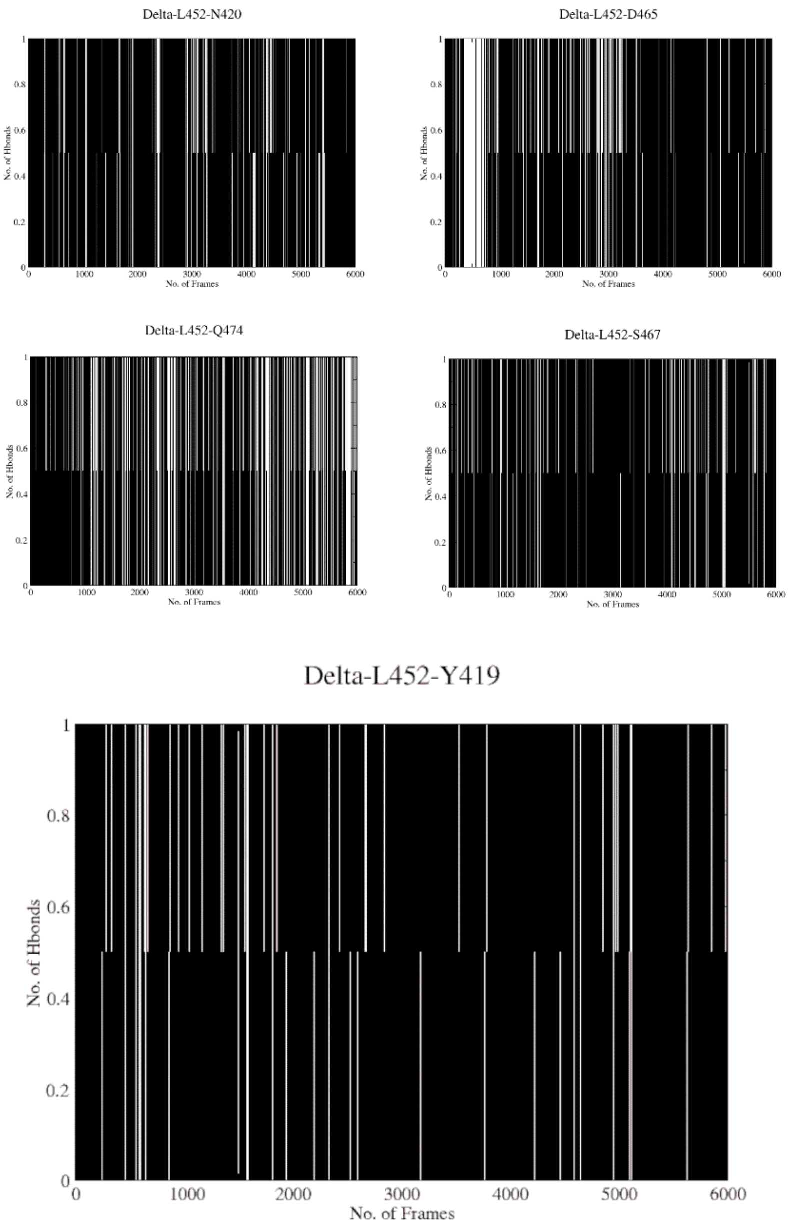

Figure S7: Hydrogen bond interactions observed during 300 ns MD simulations of RBD of variant Delta

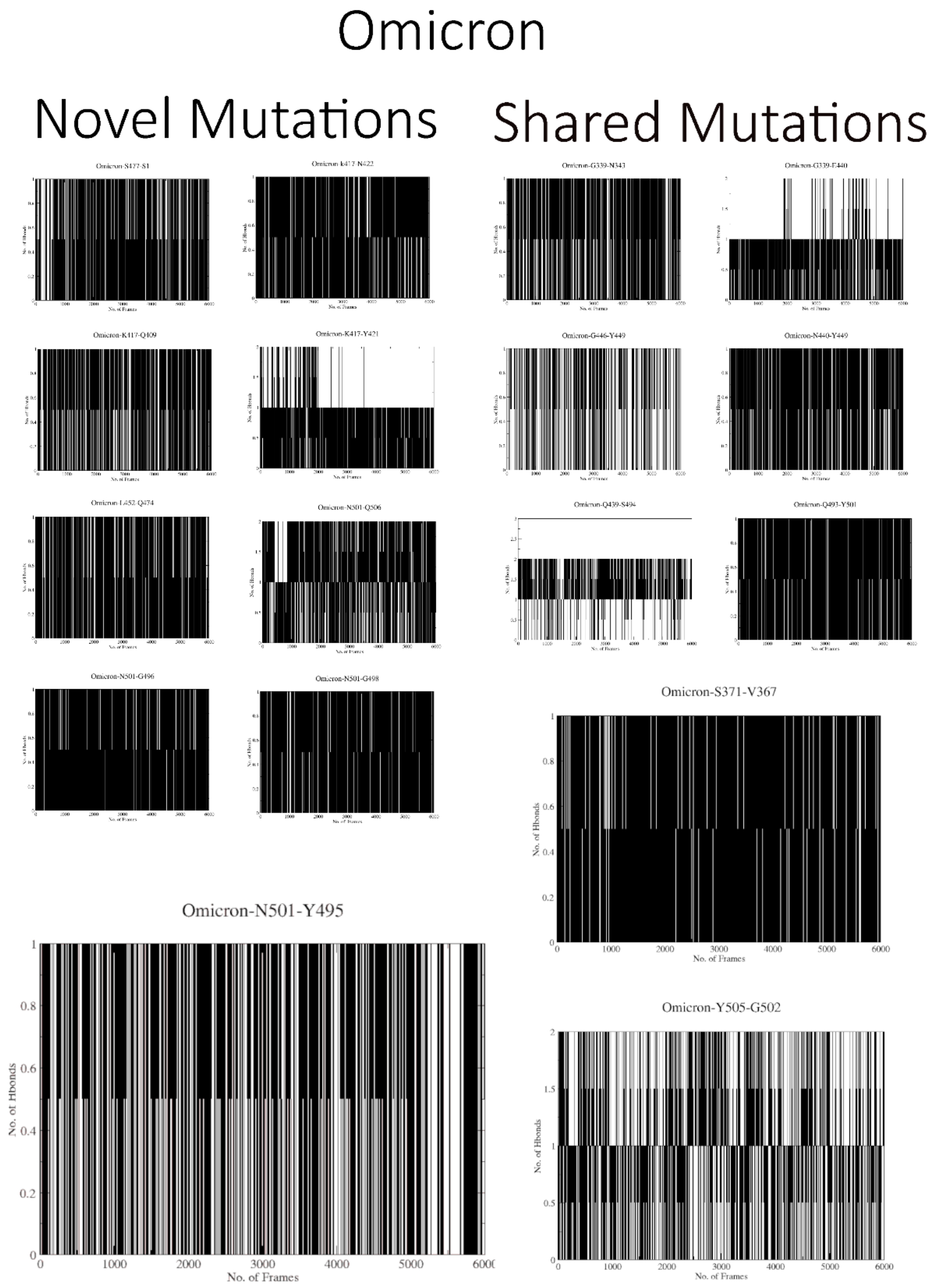

Figure S8: Hydrogen bond interactions observed during 300 ns MD simulations of RBD of variant Omicron

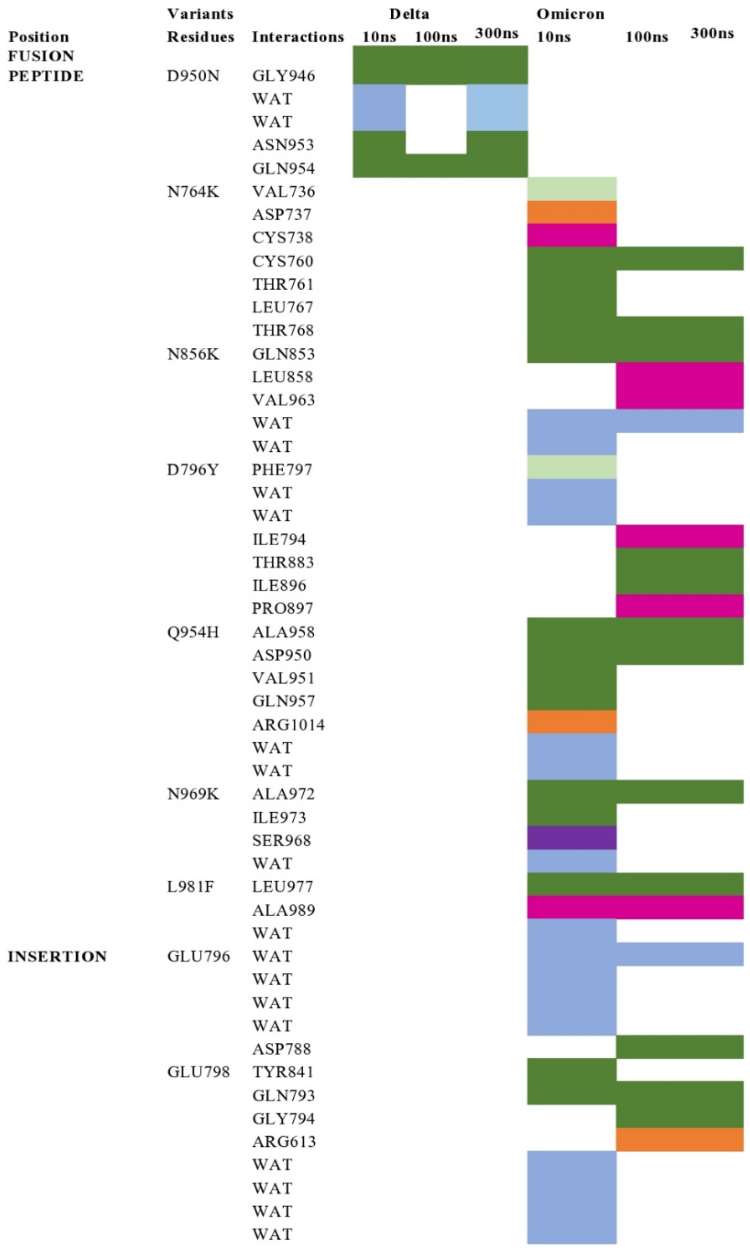

Figure S9: Intermolecular interactions of shared mutations between Omicron and Delta at fusion peptide site and EPE insertion at NTD with neighboring residues and solvent during MD simulations where (WAT) represents interactions with water molecules.

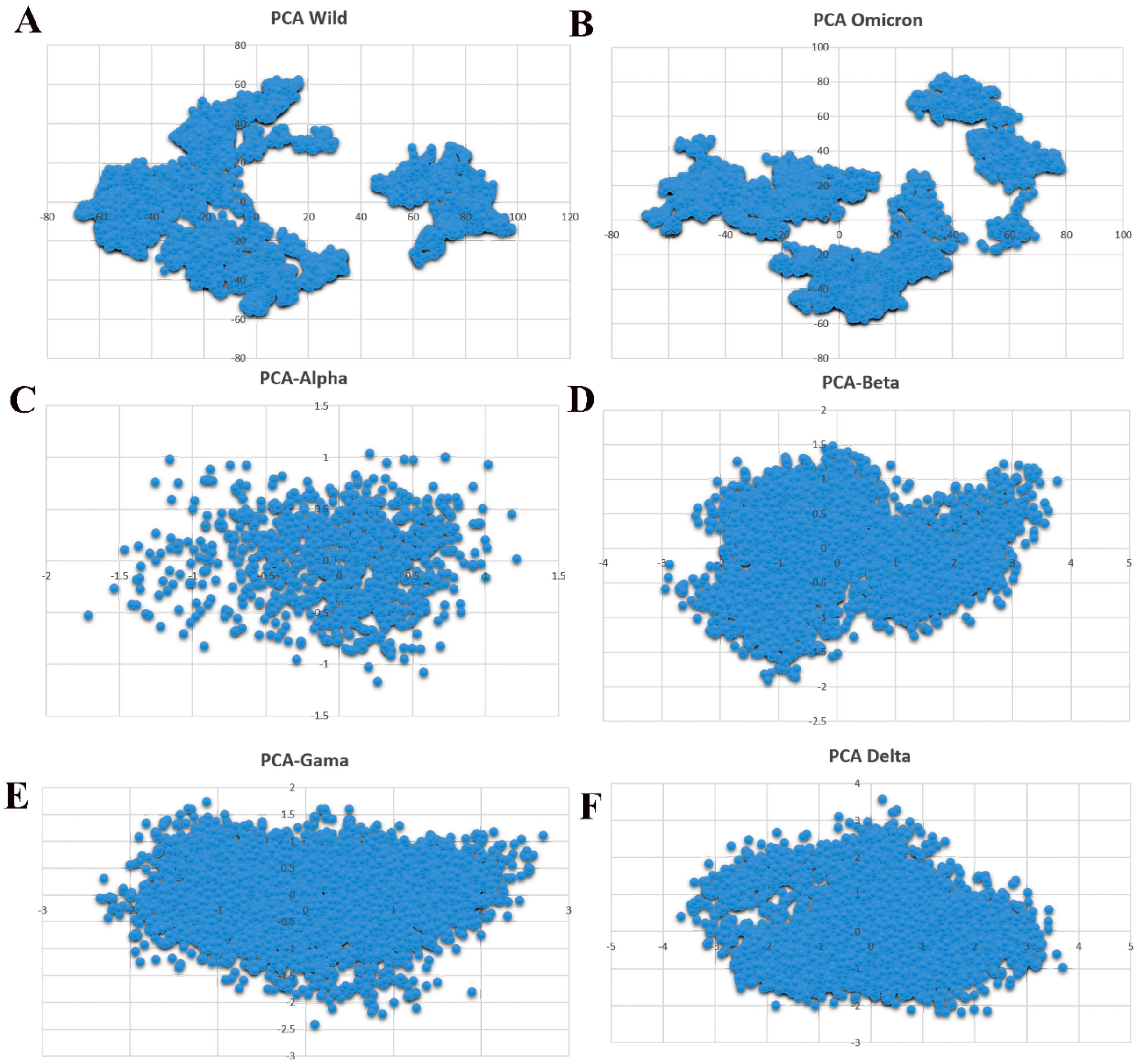

Figure S10: Overall PCA of RBDs of Alpha, Beta, Gamma, Delta, Omicron and WT exhibit varying conformer distribution along the subspace during 300 ns MD simulations each.

Table S1: Binding free energies of RBDs of all VoCs, monomers, and trimers of SARS-CoV-2 under study.

Energy Component			VDWAALS	EEL	EGB/PB	Δ G gas	Δ G solv	Δ TOTAL
RBD	Alpha	GB	-10.2126	-153.360	93.2480	-101.291	91.0950	-10.1964
		PB	-10.2126	-153.360	91.1987	-101.291	89.4401	-11.8513
	Beta	GB	-7.0551	-148.355	88.8972	-93.6664	87.2895	-6.3770
		PB	-7.0551	-148.355	87.3217	-93.6664	85.9313	-7.7351
	Gama	GB	-5.8281	-165.5843	96.7694	-105.860	95.1170	-10.7431
		PB	-5.8281	-165.5843	94.7904	-105.8601	93.4948	-12.3653
	Delta	GB	-10.3888	-112.4668	59.5686	-62.8693	57.7946	-5.0747
		PB	-10.3888	-112.4668	59.4216	-62.8693	57.9022	-4.9672
	Omicron	GB	-5.8281	-165.5843	96.7694	-105.8601	95.1170	-10.7431
		PB	-5.8281	-165.5843	94.7904	-105.8601	93.4948	-12.3653
	Wild Type	GB	-7.6616	-142.0597	80.5595	-87.4052	78.5612	-8.8440
		PB	-7.6616	-142.0597	81.1883	87.4053	79.6974	-7.7078
Monomer	Delta	GB	-6.4166	195.4275	-256.1191	253.6900	-257.4029	-3.7129
		PB	-6.4166	195.4275	-255.2718	253.6900	-256.2053	-2.5153
	Omicron with insertion EPE only	GB	-3.3381	218.077	-273.6768	272.9516	-274.6612	-1.7095
		PB	-3.3381	218.0776	-2.72.8633	272.9516	-273.6126	-0.6610
	Omicron -Fully Mutated	GB	-5.3464	120.2497	-191.0323	179.8981	-193.1353	-13.2372
		PB	-5.3464	120.24977	-189.9701	179.8981	-191.4518	-11.5537
Trimer	Omicron -Fully Mutated	GB	-2.8474	214.9573	-278.1415	285.4999	-279.2290	6.2709
		PB	-2.8474	217.9573	-278.2098	285.4999	-278.9224	6.5775
	Wild Type	GB	-7.6616	-142.0597	-268.208	299.4999	-277.9004	7.2709
		PB	-7.6616	-142.0597	-268.208	299.4999	-277.9004	7.2709

- GB refers to MMGBSA and PB refers to MMPBSA
